# Molecular and structural analysis of *Legionella* DotI gives insights into an inner membrane complex essential for type IV secretion

**DOI:** 10.1038/srep10912

**Published:** 2015-06-03

**Authors:** Takuya Kuroda, Tomoko Kubori, Xuan Thanh Bui, Akihiro Hyakutake, Yumiko Uchida, Katsumi Imada, Hiroki Nagai

**Affiliations:** 1 Graduate School of Science, Osaka University, 1-1 Machikaneyama, Toyonaka, Osaka 560-0043, Japan,; 2 Research Institute for Microbial Diseases, Osaka University, 3-1 Yamadaoka, Suita, Osaka 565-0871, Japan

## Abstract

The human pathogen *Legionella pneumophila* delivers a large array of the effector proteins into host cells using the Dot/Icm type IVB secretion system. Among the proteins composing the Dot/Icm system, an inner membrane protein DotI is known to be crucial for the secretion function but its structure and role in type IV secretion had not been elucidated. We report here the crystal structures of the periplasmic domains of DotI and its ortholog in the conjugation system of plasmid R64, TraM. These structures reveal a striking similarity to VirB8, a component of type IVA secretion systems, suggesting that DotI/TraM is the type IVB counterpart of VirB8. We further show that DotI and its partial paralog DotJ form a stable heterocomplex. R64 TraM, encoded by the conjugative plasmid lacking DotJ ortholog, forms a homo-hexamer. The DotI-DotJ complex is distinct from the core complex, which spans both inner and outer membranes to form a substrate conduit, and seems not to stably associate with the core complex. These results give insight into VirB8-family inner membrane proteins essential for type IV secretion and aid towards understanding the molecular basis of secretion systems essential for bacterial pathogenesis.

Protein secretion plays a central role in microbial pathogenesis. Many bacterial pathogens translocate effector proteins into the host cell cytoplasm using specialized secretion systems, such as type III and type IV secretion systems. These effector proteins modulate or hijack host cellular processes in order to establish infection. Type IV secretion systems (T4SSs) are ancestrally related to bacterial conjugation systems^1^,^2^,^3^. The plant pathogen *Agrobacterium tumefaciens* transports T-DNA and effector proteins into host cells using the VirB system, a prototypical T4SS. Many bacteria and conjugative plasmids encode T4SSs closely related to the VirB system, which are classified as type IVA (T4ASS)^4^. Structural studies have revealed the core complex of the conjugation system from the IncN plasmid pKM101^5^,^6^,^7^. This core complex is made of 14 molecules each of three component proteins (TraN/VirB7_pKM101_, TraO/VirB9_pKM101_ and TraF/VirB10_pKM101_). This complex spans both inner and outer membranes to form a conduit for substrate passage. Recently, a super complex containing VirB3_R388_ to VirB10_R388_, thus including the core complex, of the conjugal plasmid R388 was reported^8^.

The human pathogen *Legionella pneumophila* encodes a T4SS termed the Dot/Icm system due to its constituent *dot/icm* genes. The Dot/Icm system is essential for *Legionella* pathogenesis. Although the Dot/Icm system is closely related to the conjugation systems of IncI plasmids, such as R64 and ColIb^9^,^10^, the Dot/Icm system has little similarity to T4ASSs in gene organization and primary sequences of gene products. T4SSs closely related to the Dot/Icm system are classified as type IVB (T4BSS)^4^,^11^. We have recently reported an electron microscopic structure of the Dot/Icm T4BSS core complex containing at least five proteins DotC, DotD, DotF, DotG and DotH^12^. However, the functions of the remaining Dot/Icm proteins, most of which localize to bacterial inner-membranes, remain largely unknown.

DotI is a 23 kDa inner membrane protein essential for intracellular growth of *L. pneumophila* within mammalian and protozoan cells^13^,^14^,^15^,^16^ (Fig. S1). The gene encoding *dotI* is located immediately upstream of the genes encoding core complex component proteins DotH, DotG and DotF (Fig. 1A). DotI is conserved in all of the identified type IVB secretion systems, including the conjugation systems of R64 and related plasmids^11^. DotI has one transmembrane domain in its N-terminal region, followed by a periplasmic domain^13^. Interestingly, T4BSSs of some bacteria of the order *Legionellales*, namely *Legionella* and the aphid symbiont *Rickettsiella,* have a gene encoding DotJ immediately upstream of the gene encoding DotI. DotJ has a region with amino-acid sequence similarity to the N-terminal region of DotI (26% identity, 50% similarity), but has no periplasmic domain (Fig. 1A). Here we show that DotI and DotJ form an inner membrane complex distinct from the core complex. Structural analysis of the periplasmic domains of DotI and its R64 ortholog TraM demonstrated that DotI and TraM are structural homologs of the T4ASS protein VirB8. The cellular localization of DotI is clearly different from polar localization of core complex components DotG and DotF. Collectively, DotI participates in the assembly of a pivotal T4SS complex distinct from the core complex.

## Results

### DotI and DotJ are dependent on each other for robust expression

**Figure 1:**
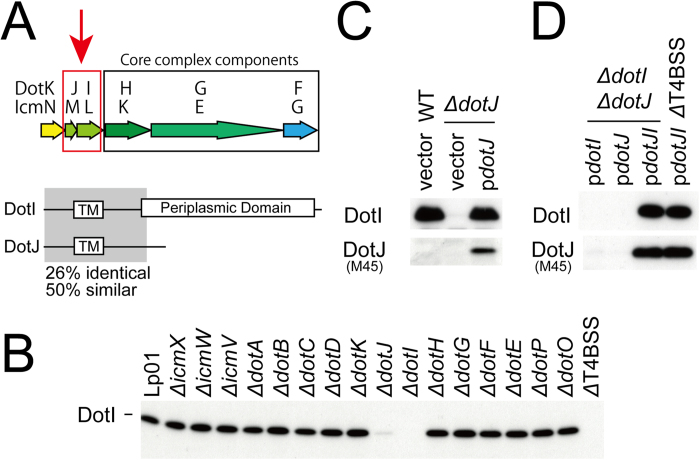
Genetic interaction between DotI and DotJ. (**A**) Schematic drawings of genes encoding DotK, DotJ, DotI and core complex components DotH, DotG and DotF (top), and domain organization of DotI and DotJ (bottom). (**B**) Levels of DotI in lysate from various deletion mutants of *L. pneumophila*. Dot/Icm genes deleted are indicated above the Western immunoblot using DotI antibody. (**C**) Reduction of DotI level in *ΔdotJ* strain is complemented by supplying M45-DotJ from a plasmid. Western immunoblots using DotI and M45 antibodies are shown. (**D**) Supplying DotI and M45-DotJ *in trans* is enough for robust expression of DotI and DotJ in deletion strains lacking both DotI and DotJ (*ΔdotI ΔdotJ*) or the entire set of *dot/icm* genes (*Δ*T4BSS).

For proteins that form complexes, individual components often show reduced stability in the absence of their interaction partners. Vincent and Vogel examined the steady-state levels of Dot/Icm proteins in various in-frame deletion strains of *dot/icm* genes^17^. The results suggested interactions between a number of Dot/Icm proteins, including ones among core complex components DotC, DotH and DotG. To gain insight into the binding partner of DotI, we examined DotI levels in various *dot/icm* deletion strains (Fig. 1B). DotI levels were not affected in most of the *dot/icm* deletion strains examined. In contrast the DotI level was significantly reduced in a deletion strain unable to produce DotJ. Because the gene encoding DotJ is located immediately upstream of the gene encoding DotI (Fig. 1A), the reduction in DotI in the absence of DotJ might be attributed to polar effect of the *dotJ* deletion. To test this possibility, we examined DotI levels in *dotJ* deletion strains carrying plasmids encoding either M45-epitope tagged *dotJ* or the empty vector control (Fig. 1C). Expression of DotJ from the complementing plasmid was confirmed using an anti-M45 antibody. Supplying DotJ *in trans* was sufficient to restore DotI to wild-type levels, which clearly excludes the possibility of polar effects.

Next, we asked whether DotI is required for robust expression of DotJ. To test this, we examined M45-tagged DotJ levels expressed from plasmids in a double deletion strain lacking both *dotI* and *dotJ* (Δ*dotJI*) (Fig. 1D). The DotJ level in the strain not producing DotI (Δ*dotJI* p*dotJ*) was clearly lower than in the strain producing both DotI and DotJ (Δ*dotJI* p*dotJI*). Consistent with the original finding, the DotI level in the strain unable to produce DotJ (Δ*dotJI* p*dotI*) was remarkably lower than in the strain producing both DotI and DotJ (Δ*dotJI* p*dotJI*). Taken together, robust expression of DotI and DotJ in *L. pneumophila* appears to be dependent on production of both proteins. Furthermore, robust expression of DotI and DotJ does not require other Dot/Icm proteins, because DotI and DotJ levels were not affected by the absence of the other Dot/Icm proteins (ΔT4BSS p*dotJI*). These results suggest that DotI and DotJ interact with each other and form a complex within *L. pneumophila*.

### DotI and DotJ form a hetero-complex in *L. pneumophila*

**Figure 2:**
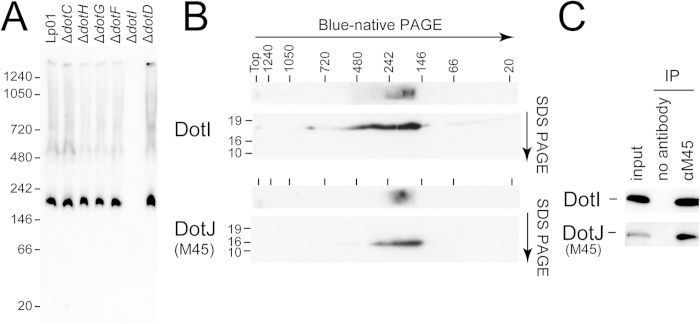
DotI and DotJ form a stable complex. (**A**) BN-PAGE analysis of *L. pneumophila* lysates from wild-type and deletion strains. Deleted genes are indicated above the Western immunoblot using DotI antibody. (**B**) Two-dimensional PAGE analysis of *L. pneumophila* producing M45-DotJ. Samples were separated by BN-PAGE, and then gel slices were subjected to second-dimensional SDS-PAGE. Western images of aligned one (top) and two (bottom) -dimensional PAGEs using DotI and M45 antibodies are presented with positions of molecular weight markers (in kDa). (**C**) Co-immunoprecipitation of DotI and M45-DotJ. Lysates from *L. pneumophila* producing M45-DotJ were subjected by immunoprecipitation with or without M45 antibody, followed by Western immunoblotting analysis using DotI or M45 antibodies.

Native polyacrylamide gel electrophoresis (PAGE) analysis is often employed to evaluate complex formation in a native state. We took advantage of the blue-native polyacrylamide gel electrophoresis (BN-PAGE) system to explore formation of complexes containing DotI in *L. pneumophila. L. pneumophila* proteins were extracted with a buffer containing a non-ionic detergent n-dodecyl-β-D-maltoside (DDM) and subjected to BN-PAGE analysis followed by Western immunoblotting using an anti-DotI antibody (Fig. 2A). A strong signal was detected with an apparent molecular weight between 146 and 242 kDa. No signal was detected in extracts from the *dotI* deletion strain (Δ*dotI*). These results indicate that wild-type *L. pneumophila* produces a complex containing DotI, which has an apparent molecular weight of ~200 kDa. In addition, the amounts and size of the DotI complex was not affected by the absence of core complex components DotC, DotD, DotF, DotG and DotH, suggesting that the DotI complex is formed independently of the core complex (Fig. 2A). This is consistent with the fact that robust expression of DotI and DotJ does not require the other Dot/Icm proteins (Fig. 1D).

Because the theoretical molecular weight of DotI is 23 kDa, the ~200 kDa DotI complex must contain multiple DotI molecules and/or other proteins like DotJ. To test whether the DotI complex contains DotJ, we conducted two-dimensional PAGE analysis of strains producing M45-tagged DotJ (Fig. 2B). The DotI complex was separated by molecular weight in native condition using BN-PAGE, followed by secondary separation in denaturing conditions using SDS-PAGE. In both immunoblots using either anti-DotI or anti-M45 antibodies we detected a ~200 kDa complex. This result strongly suggests that the ~200 kDa complex contains both DotI and DotJ. To further test whether DotI and DotJ form a complex, we conducted co-immunoprecipitation analysis using *L. pneumophila* producing M45-DotJ. DotI was co-precipitated with M45-DotJ only in the presence of the anti-M45 antibody, which indicates the interaction between DotI and DotJ occurs in bacterial cells (Fig. 2C).

### Characterization of the DotI-DotJ complex

**Figure 3:**
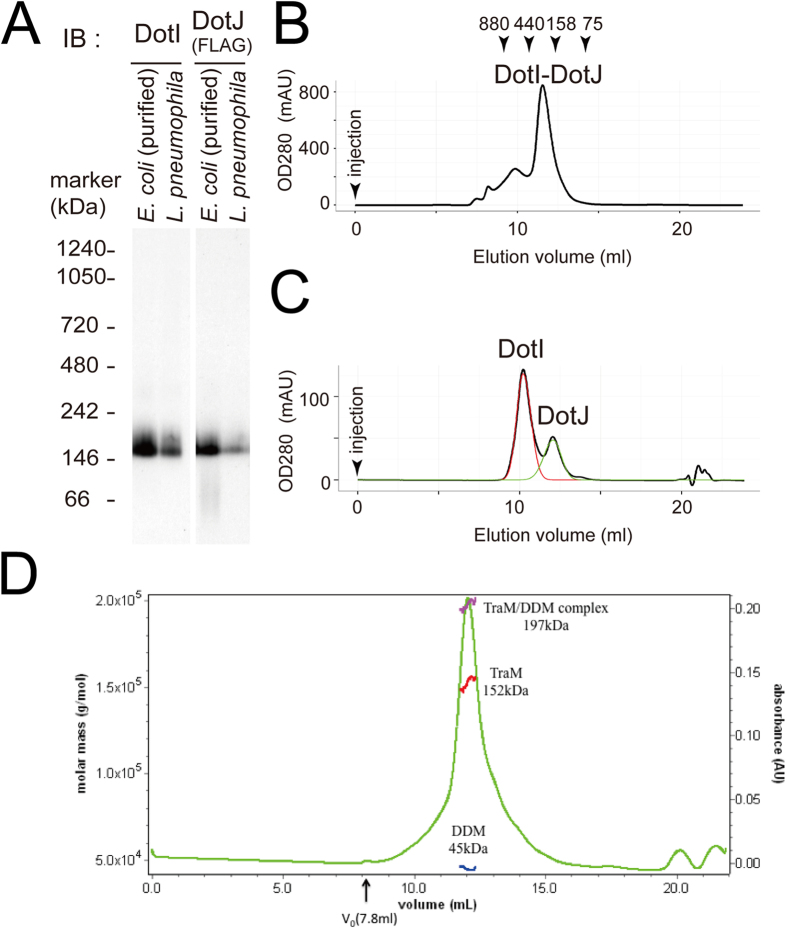
Characterization of DotI-DotJ and TraM complexes. (**A**) BN-PAGE analysis of DotI-DotJ complex purified from *E. coli* producing DotI and Strep-3xFLAG-DotJ, and that formed in *L. pneumophila* producing DotI and Strep-3xFLAG-DotJ. Flag antibody was used for DotJ detection. (**B**) Size exclusion chromatogram of the purified DotI-DotJ complex. Elution points of some standard proteins are also shown. (**C**) Size exclusion chromatogram of pooled peak fractions in panel B. Mobile buffer contains 6M Urea and 1mM DTT to dissociate DotI and Strep-3xFLAG-DotJ. Red and green lines indicate computationally separated chromatograms for DotI and Strep-3xFLAG-DotJ, respectively (see text). (**D**) Size exclusion chromatogram of His-TraM complex purified from *E. coli*. Elution profile of the complex associated with detergent DDM is shown with the molecular weight estimated by MALS. The molar masses of the TraM-DDM complex, TraM, and DDM throughout the elution peak are indicated by the magenta, red and blue lines, respectively.

Based on the above findings, we hypothesized that DotI and DotJ form a stable complex independently from other *L. pneumophila* proteins. To test this hypothesis, we constructed an *Escherichia coli* strain producing DotI and Strep-tagged DotJ. The majority of DotI and DotJ expressed in *E. coli* was found in the crude membrane fraction (Fig. S2). After solubilization with DDM, complexes containing DotJ were affinity purified using Streptactin resin (Fig. S2). The DotI-DotJ complex purified from *E. coli* showed an indistinguishable mobility in BN-PAGE analysis from that observed with Strep-DotJ-producing *L. pneumophila* (Fig. 3A), suggesting the purified complex has the same molecular weight as the complex formed natively in *L. pneumophila*.

The purified complex eluted at a volume corresponding to >158 and <440 kDa in size exclusion column chromatography (Fig. 3B). The estimated molecular weight of the major peak agrees well with the BN-PAGE results. Nevertheless, this apparent molecular weight may be an overestimation because the DotI-DotJ complex is bound to detergent molecules. Size exclusion chromatography-multi angle light scattering (SEC-MALS) analysis of the complex was also conducted, however, this analysis failed due to the presence of higher-order aggregates which overlapped with the main peak in size exclusion chromatography. To determine the stoichiometry of the complex, we collected peak fractions from size exclusion column chromatography and pooled peak fractions were re-analyzed in the presence of chaotropic and reducing agents to separate DotI and DotJ (Fig. 3C). Peaks representing each protein were computationally deconvoluted assuming the biGaussian model (Fig. 3C, red and green lines for DotI and DotJ, respectively) and the molecular ratio was estimated as 1.87 DotI : 1 DotJ using extinction coefficients of each protein.

### Oligomeric state of R64 TraM

The results of native PAGE and size exclusion chromatography suggested that the DotI-DotJ complex forms a higher order complex. The conjugal plasmid R64 has no counter part of DotJ, thus TraM is expected to assemble into a homomultimer. To elucidate the oligomerization of TraM, we expressed and purified TraM using an *E. coli* expression system, and conducted SEC-MALS measurement. The purified TraM eluted as a single peak with following weak shoulders (Fig. 3D). We then separated the contributions of protein and detergent (DDM) to the scattering profile, and calculated the molecular weight. The estimated molecular weights of TraM and DDM are 152 kDa and 45 kDa, respectively. Considering the molecular weight of TraM (26 kDa) and DDM (0.5 kDa), TraM in solution is in a hexameric state with about 90 detergent molecules.

### Crystal structures of the DotI and TraM periplasmic domain

**Figure 4:**
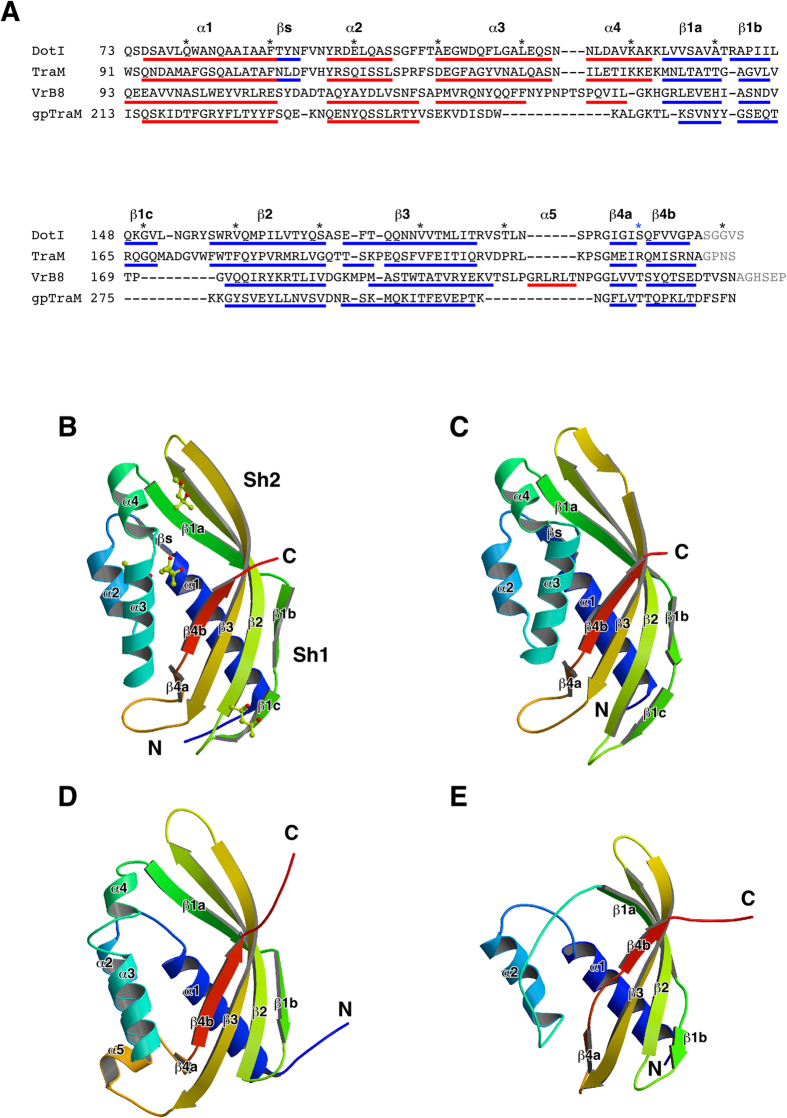
Crystal structures of DotIC and its structural homologs. (**A**) Structure based alignment of DotI, R64 TraM, *A. tumefaciens* VirB8 (PDB ID 2CC3) and *E. faecalis* TraM (gpTraM) (PDB ID 4EC6). (B–E) Cα ribbon representation of DotI_**C**_ (**B**), R64 TraM_**C**_ (**C**), *A. tumefaciens* VirB8 (PDB ID: 2CC3) (**D**) and *E. faecalis* TraM (PDB ID: 4EC6) (**E**). (**B**) The MPD molecules bound to DotI_**C**_ are indicated by the ball and stick model.

To gain insight into the role(s) of DotI in type IVB secretion, we determined the crystal structures of the C-terminal periplasmic domains of DotI (residues 73-212, DotI_C_) and its conjugative plasmid R64 ortholog TraM (residues 91-230, TraM_C_). DotI_C_ crystallized in two distinct forms (Form I and Form II) under different conditions. The structure model of Form I (space group: *P*2_1_2_1_2) was refined to a resolution of 2.2 Å with the working R and the free R factor of 19.1% and 23.5%, respectively, and Form II (space group: *I*432) to a resolution of 3.5 Å with the working R and the free R factor of 17.9% and 21.6%, respectively. The X-ray data collection and refinement statistics are summarized in Table S2. The five most C-terminal residues were invisible due to disorder. Form I and Form II contain eight and four molecules in its asymmetric unit, respectively, and the structures of these molecules are almost identical. DotI_C_ is a single domain protein composed of four helices (α1 - α4) in one half and one β-sheet in the other half (Fig. 4A,B). The sheet is highly twisted at its center with an interruption in β1, therefore can be divided into two parts, Sh1 and Sh2. Sh1 consists of β1b, β1c, the N-terminal half of β2, the C-terminal half of β3, β4a and β4b, and forms a hydrophobic core with helix α1. Sh2 comprises βs, β1a, the C-terminal half of β2 and the N-terminal half of β3, and forms a large cleft with α3 and α4. The cleft of the molecule in the Form I crystal is filled by 2-methyl-2,4-pentanediol (MPD) molecules, which is included in the mother solution for the Form I crystal.

The structure of TraM_C_ was determined at 1.5 Å resolution with the working R and the free R factor of 18.0% and 20.7%, respectively (Table S2). The crystallographic asymmetric unit contains a single molecule. As expected from the considerable amino-acid sequence similarity between DotI_C_ and TraM_C_ (30% identity, 54% similarity), the structure of TraM_C_ (Fig. 4C) is similar to that of DotI_C_ (Fig. 4B). They can be superimposed with an rmsd (root mean square deviation) for corresponding Cα atoms (106 of 137 atoms) of 0.876 Å. The remarkable structural difference is the position of α3. α3 of TraM_C_ leans to Sh2, thereby closing the cleft.

The DaliLite database search^18^ showed that DotI_C_ has striking structural similarity (Z-score 13.4 to 14.2) to the periplasmic domains of *A. tumefaciens* and *Brucella suis* VirB8, an essential inner membrane protein of T4ASSs^19^,^20^ (Fig. 4D), although the sequence identity is only 9.0% and 10.7%, respectively. Addition to these, many similar structures of VirB8 proteins from gram-negative *Bartonella* sp. (PDB ids 4JF8, 4KZ1, 4LSO, 4MEI, and 4NHF) and *Richettsia typhi* (4O3V) were published in PDB database. The arrangement of the VirB8 secondary structure is essentially the same as that of DotI_C_ except for α3 and α5 (Fig. 4A,D). α3 of VirB8 is almost anti-parallel to α2, thus the cleft between Sh2 and α3-α4 opens wider than the cleft of DotI_C_. The corresponding region of α5 is an extended loop structure in DotI_C_. The DotI_C_ structure also resembles (Z-score 10.4 to 10.8) recently published crystal structures of TcpC and TraM from the conjugative plasmids pCW3 and pIP501 found in the gram-positive bacteria *Clostridium perfringens* and *Enteroccocus faecalis*, respectively^21^,^22^ (Fig. 4E). In summary, the protein domains described here constitute a widely conserved structural domain in T4SSs both from gram-negative and gram-positive bacteria.

### DotI_C_ forms a ring structure

**Figure 5:**
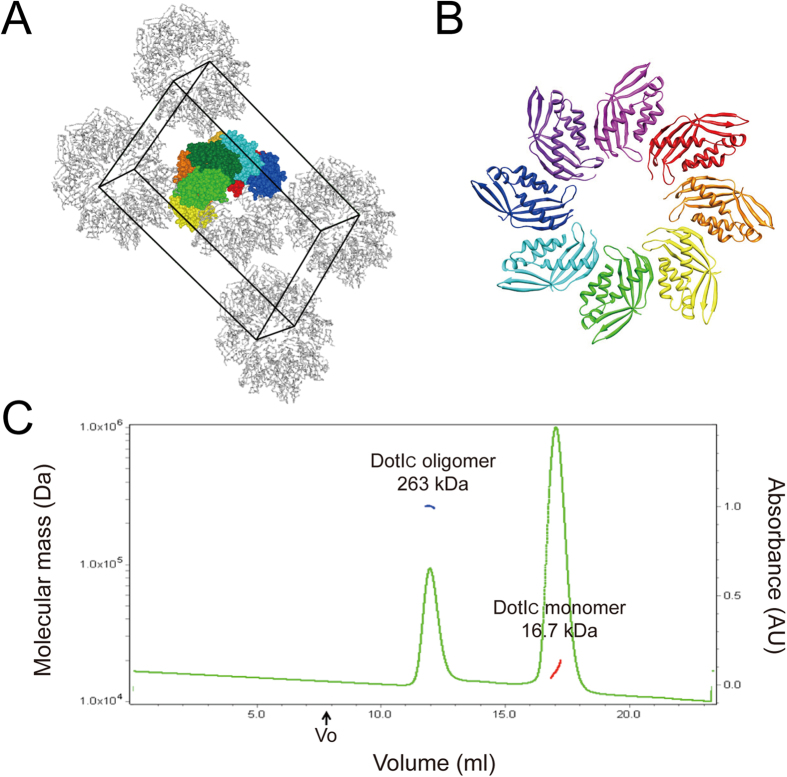
DotIc forms ring-like oligomers in crystals. (**A**) Molecular packing of DotIc in the *P*2_1_2_1_2 crystal. The molecules in an asymmetric unit are shown by ball models with different colors, and other symmetry related molecules are indicated by Ca backbone trace. The unit cell is shown by black line. (**B**) The ribbon diagram of the DotIc octamer ring in the crystal. (**C**) Size exclusion chromatogram of DotI_C_ in a crystallization condition. Elution profile of DotI_C_ oligomer and monomer is shown with the molecular weight estimated by MALS. The molar masses corresponding to the oligomer and the monomer throughout the elution peaks are shown by the blue and red lines, respectively.

DotI_C_ forms a stack of two octameric rings in the Form I crystal (Fig. 5A,B). This asymmetric unit contains eight DotI_C_ molecules that form a head-to-head stack of two half rings composed of four monomers each. The crystallographic two-fold axis at the center of the half rings produces the two octameric rings. The octameric rings are also present in the Form II crystal. The four molecules in the asymmetric unit of Form II are arranged as a stack of a quarter of the ring, and the crystallographic four-fold axis produces a stack of two octameric rings. The oligomerization of DotI_C_ is also observed in solution. Although DotI_C_ is in a monomeric state during purification, an oligomeric peak appears in size exclusion chromatography under the crystallization condition (Fig. 5C). SEC-MALS measurement showed that the apparent molecular weight of the oligomer is 263 kDa, which is consistent with the molecular weight of the stack of two octameric rings. This evidence suggests that octameric ring formation is not a crystal packing artifact, but instead an intrinsic property of the DotI_C_ molecule.

**Figure 6:**
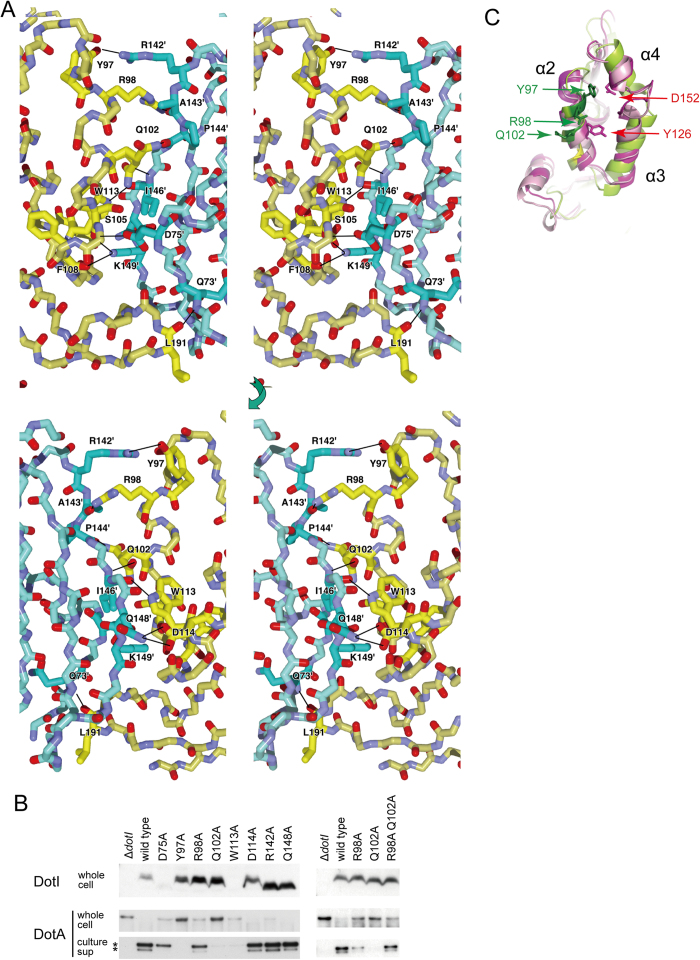
Mutational analysis of the putative DotI ring. (**A**) Close up stereo views of the subunit interface in the DotIc octameric ring. The top panel is viewed from the octamer-octamer interface, and the bottom panel from the opposite side of the top. The two subunits are colored in yellow and cyan. The residues involved in the subunit interaction are drawn with side chain and highlighted with vivid color. Inter subunit hydrogen bonds are indicated by black lines. (**B**) Effects of DotI mutations in DotA secretion. *L. pneumophila* producing wild-type or indicated DotI mutants were analyzed. DotI levels in whole cell lysates and DotA levels in whole cell lysates and culture supernatants are shown. Secreted DotA is processed by an extracellular MspA protease, which gives rise to a doublet of 70–80 kDa as major products 23, as indicated by the asterisks. (**C**) Molecular surface formed by α2 and α4 helices. Positions of Tyr-97, Arg-98 and Gln-102 of DotI (green), Tyr-126 and Asp-152 of *B. suis* VirB8 (magenta) are shown.

Inter-subunit hydrophilic interactions between main chain atoms and between side chain and main chain atoms mainly contribute to the octamer ring formation. The subunit interface is composed of α2, α3 and the loop connecting β3-β4 on one side, and β1b, β1c and the N-terminal region on the other side (Fig. 6A). The main chain oxygen of Leu-191 hydrogen-bonds with the main chain NH of Gln-73’ (‘denotes the residues of the neighboring molecule). Main chain oxygen atoms of Phe-108 and Ser-105 form a hydrogen-bonding network with the side chain atoms of Lys-149’ and Asp-75’. The indol NH of Trp-113 and the guanidino group of Arg-98 interact with the main chain oxygen atoms of Ile-146’ and Ala-143’, respectively. The side chain of Gln-102 adopts two conformations, one interacts with the main chain NH of Ile-146’ and the other with the main chain carbonyl oxygen of Pro-144’. Interactions between side chain atoms were found only at the edge of the interaction surface (Asp-114 interacts with Gln-148’, and Tyr-97 with Arg-142’.) These interactions, however, seem to contribute less towards stabilization of the ring, because the orientation of the side-chains deviates from the ideal orientation for hydrogen-bonding.

To elucidate the significance of these subunit interactions for T4BSS function, we made alanine substitutions at residues with side chains that supposedly participate in the interaction and examined secretion of DotA into culture supernatants, which is a Dot/Icm T4BSS-dependent process^23^ (Fig. 6B). The levels of DotA secretion in the mutant strains (Fig. S6B) correlate well with intracellular growth within *Acanthamoeba castellanii* (Fig. S3), supporting the notion that DotA secretion is a good measure to monitor Dot/Icm T4BSS function. Eight single substitution mutants (D75A, Y97A, R98A, Q102A, W113A, D114A, R142A and Q148A) were prepared and expressed in the Δ*dotI* mutant of *L. pneumophila*. Six mutant proteins except D75A and W113A were expressed at wild-type levels. It should be noted that residual amounts of D75A mutant protein may account for partial DotA secretion from the strain producing the mutant protein. Among them, three mutants (D114A, R142A and Q148A) showed no defect in DotA secretion, suggesting that the putative inter-subunit side-chain interactions at the edge of the interaction surface are not essential for secretion. In contrast, Y97A and Q102A mutants showed severe secretion defects. The R98A mutant showed a subtle but significant defect in DotA secretion. Tyr-97, Arg-98 and Gln-102 are aligned on the same surface of α2 (Fig. 6C), indicating that this surface plays an important role in protein secretion. These results are consistent with the hypothesis that DotI forms oligomers under physiological conditions in a Tyr-97-, Arg-98- and Gln-102- dependent manner, however, we found that an R98A Q102A double mutant somehow restores DotA secretion (Fig. 6B), which is not well explained by this hypothesis.

### DotI forms distributed foci in *L. pneumophila*

**Figure 7:**
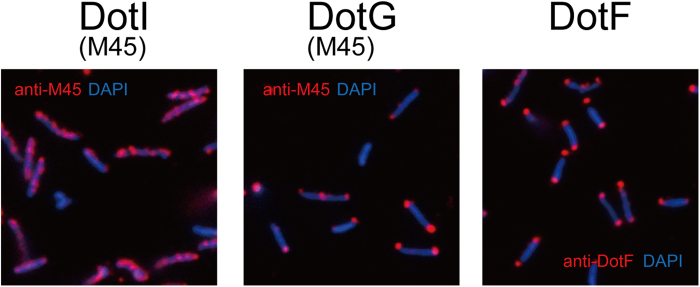
Cellular localization of DotI, DotG and DotF. *L. pneumophila* strains were fixed with formaldehyde and attached to cover slips. Fixed bacteria were treated with lysozyme followed by methanol to permeabilize bacterial peptideglycan layer and membranes, and then stained with indicated antibodies and DAPI. Localization of Dot proteins are shown in red, bacteria are shown in blue.

It was reported that *A. tumefaciens* VirB8 and the core complex component proteins VirB7, VirB9 and VirB10 localize to bacterial poles^24^. More recently using deconvolution microscopy, GFP-VirB8 and some GFP-tagged substrate proteins were reported to form helical arrays around the circumference of bacterial cells after induction of *vir* genes^25^. To clarify the localization of DotI-DotJ and core complexes in *L. pneumophila*, we conducted immunofluorescence microscopy analysis of *L. pneumophila* strains producing either M45-DotI or M45-DotG grown in laboratory medium to infection-competent stationary phase (Fig. 7). We first confirmed that the expression levels of M45-DotI and M45-DotG are equivalent to their counterparts in the isogenetic wild-type strain (Fig. S4A,B), and that adding an M45-tag to DotI or DotG does not affect intracellular growth of *L. pneumophila* (Figs. S1 and S4C). M45-DotI was found to form foci distributed throughout bacterial cells. This localization pattern is similar to that reported for GFP-VirB8 by Aguilar *et al.*^25^, rather than the polar localization reported by another group. In contrast two core complex component proteins M45-DotG and DotF were mostly found at both poles of bacterial cells. These results demonstrate that the majority of DotI-DotJ complex does not stably interact with the core complex in infection-competent *L. pneumophila*.

## Discussion

Here we demonstrated that the periplasmic domains of *L. pneumophila* DotI and its R64 ortholog TraM share striking structural similarity to that of the T4ASS component VirB8. Structural homologs of VirB8 were found in T4S/conjugation systems both from gram-negative and -positive bacteria^19^,^20^,^21^,^22^. Overall domain architecture of T4BSS DotI (Fig. 1A) and TraM is very similar to that of T4ASS VirB8, suggesting that DotI/TraM is the functional counterpart of T4ASS VirB8. In contrast, domain organization of pCW3 TcpC and pIP501 TraM from gram-positive bacteria is apart from that of VirB8-like proteins from gram-negative bacteria^21^,^22^. The differences may be due to the altered necessities of gram-negative and -positive T4SSs and/or to fundamental differences between the gram-negative and -positive bacterial cell surface structure.

DotI forms a stable complex with its partial homolog DotJ. In *L. pneumophila* both DotI and DotJ are essential proteins for T4S (Fig. S1). Whereas DotI is conserved in all T4BSSs, including conjugation systems, DotJ is only conserved in the Dot/Icm systems of *Legionella* and *Rickettsiella*. Genomic information so far available indicates that all bacteria of the order *Legionellales* encode Dot/Icm-like T4BSSs on their chromosome, suggesting that the Dot/Icm system is acquired by a common ancestor and vertically transmitted thereafter^11^. *Coxiella burnetii*, another bacterium of the order *Legionellales,* does not encode DotJ but instead carries duplicated DotI genes. The acquisition of DotJ or duplication of DotI may be related to the needs for translocation of effector proteins into host cells, because all of *Legionellales* bacteria are intracellular pathogens or endosymbionts.

The exact oligomeric state of the DotI-DotJ complex remains to be clarified, whereas TraM clearly forms a hexameric homo-oligomer. In contrast DotI_C_ forms octameric rings in crystals and in solutions used to obtain crystals. The mutational analysis of DotI_C_ suggests that some residues involved in subunit interaction in the octamer are important for T4BSS function, but the presence of the DotI octamer in the T4BSS complex is still obscure. To date several crystal structures of VirB8 structural homologs, including*A. tumefaciens* VirB8, *E. faecalis* TraM and *L. pneumophila* DotI, have been reported to form oligomers. Although the protomer structures are closely related to each other (Fig. 4), the numbers of protomer in each oligomer and the way of inter-protomer contact is remarkably different (Fig. S5). These findings imply that VirB8 structural homologs have a propensity to form oligomers under crystallization conditions, although the oligomers found in the crystals do not necessarily represent their native state in bacterial cells. Their N-terminal regions containing a *trans*-membrane domain, which are missing in proteins used for crystallization studies, are likely to play a pivotal role in oligomer formation in bacterial cells. This underlines the importance of examination of native complexes, rather than soluble fragments, to explore the nature of VirB8 structural homologs.

Several lines of evidence suggest that the T4ASS component VirB8 interacts with substrate proteins: VirB8 can be crosslinked to substrate nucleic acid^26^ and colocalized with GFP-substrate proteins^25^ in *A. tumefaciens*. A simple model to explain its importance in T4S and its ability to interact with substrates would be that VirB8 and core complex form a functional super complex in which VirB8 makes a conduit through which substrates traverse. In *L. pneumophila* grown in laboratory medium, the majority of DotI does not localize to bacterial poles, which is where the core complex components DotG and DotF accumulate (Fig. 7). Thus the majority of DotI-DotJ complex is not spatially available for super complex formation at cell poles. Consistently, we failed to detect high molecular weight complexes containing both DotI-DotJ and core complexes from *L. pneumophila* membrane fractions (Fig. 2A, Fig. 3A), and we failed to pull-down the core complex components together with DotI or DotJ efficiently from bacterial lysates (TKub, unpublished). Along the same lines, the core complex of the T4ASS conjugal plasmid pKM101 was purified from *E. coli* strain producing VirB7, VirB8, VirB9 and VirB10, whereas VirB8 was excluded from the purified core complex^6^. One caveat to these arguments, however, is that DotI-DotJ and core complexes might assemble together dynamically on demand.

Recently, Low *et al.* reported an electron microscopic structure of the R388 T4ASS VirB3–VirB10 complex^8^, in which the core complex is connected through a central stalk to two hexameric barrel-like inner membrane complexes containing VirB4. They also estimated location and stoichiometry of each component protein in the complex. Although the position of VirB8 in the complex is still unclear, the stoichiometry of VirB8 was estimated as twelve per VirB3–VirB10 complex using a radio-iodination technique, suggesting VirB8 may be located on the top of each hexameric barrel of VirB4^8^. Although no counterparts of VirB3, VirB5 and VirB6 were so far found in T4BSS, the structural similarity of DotI/TraM to VirB8 implies that a similar inner membrane complex may be present in T4BSS. Our SEC-MALS result showing that full-length TraM forms a hexameric complex is consistent with this idea. In contrast, the DotI_C_ octamer observed under crystallization conditions does not fit with the T4ASS super complex. Taking into account that (i) DotI forms a complex with DotJ with a molecular ratio of ~2:1, and (ii) only a subset of T4BSSs and no T4ASS contain DotJ, one possible explanation is that DotI and DotJ may form a hetero-hexamer containing four DotI and two DotJ molecules (DotI_4_DotJ_2_; theoretical complex mass is 115kDa) and form a VirB8-like inner membrane complex. The VirB8-like inner membrane complex may transiently interact with the core complex to form transport-active super complex. In this case the VirB8-like complex is likely to function as an inner-membrane conduit. Alternatively, the structure of the DotI-DotJ-containing inner membrane complex of Dot/Icm T4BSSs diverges from the T4ASS architecture. Elucidation of the exact function of the DotI-DotJ/VirB8 complex and mechanism of T4S awaits future studies.

## Methods

### Bacterial strains, plasmids and culture

Bacterial strains and plasmids used in this study are provided in Table S1. *L. pneumophila* strains were grown on charcoal-yeast extract (CYE) or in ACES buffered yeast extract (AYE) media^27^. Chloramphenicol or kanamycin was added to the media (10 μg/ml or 10 μg/ml, respectively) when required.

### Antibodies

DotI antibody was raised against purified DotI_C_ and affinity purified. Antibodies against DotF and M45 epitope were described previously^28^. Anti-FLAG antibody was purchased from Sigma-Aldridge (F3165).

#### Preparation of *L. pneumophila* membrane fractions

*L. pneumophila* membrane fractions were prepared by the sucrose step gradient centrifugation described previously^29^ with some modifications. *L. pneumophila* strains were grown in 25 ml (10 test tubes with 2.5 ml each) of AYE for 24 hours at 37 °C and harvested by low speed centrifugation (7,000 g × 10 min at 4 °C). The pellet was suspended with 5 ml of cold 20% (w/w) sucrose, 10 mM HEPES-OH pH 7.4. DNaseI and RNaseA were added (final 10 μg/ml each) and the bacterial cells were disrupted by French Pressure cell press in the presence of 1 mM phenylmethylsulfonyl fluoride (PMSF). Unlysed cells were removed by low speed centrifugation (3,000 g × 15 min at 4 °C) and recover the supernatant. EDTA was added to the lysate (final 5 mM). 1.6 ml of the lysate was applied on the two-step sucrose gradient of 0.8 ml of 60% (w/w) and 2.4 ml of 25% (w/w) sucrose in 10 mM HEPES-OH pH 7.4. The samples were ultracentrifugated in SW55Ti rotor (Beckman) with 150,000 x g for 3.5 hours at 4 °C. The crude membrane fraction was visible as an opaque layer at the boundary of 60% (w/w) and 25% (w/w) sucrose. The membrane fraction was carefully recovered and diluted with cold 10 mM HEPES-OH pH 7.4 and 5 mM EDTA solution to a final volume of 1.2 ml.

#### Blue native-polyacrylamide gel electrophoresis

Blue native-polyacrylamide gel electrophoresis (BN-PAGE) was carried out according to the manufacture’s recommendation using 4-16% or 3-12% gradient gels from NativePAGE Novex Bis-Tris Gel System (Invitrogen) with some modifications. Briefly, membrane fraction or membrane protein samples were mixed with 2x Sample buffer (50 mM Bis-Tris pH 7.2, 750 mM 6-aminocapoic acid, 50 mM NaCl, 10% (w/v) glycerol, 0.1% (w/v) TritonX-100, 1% (w/v) DDM and 0.001% (w/v) PonceauS in final concentration). After incubation with mild agitation for 2 hours at 4 °C, 5% (w/v) Coomassie G-250 solution was added to the sample to give final concentration of 0.25% (w/v). The samples (equivalent to 50-70 mOD_600_ unit of cell mass per lane) were loaded on the gels. Cathode and Anode buffers were prepared by diluting 20x Native PAGE Running buffer (Invirtogen) and 20x Native PAGE Cathode Buffer Additive (Invitrogen) with distilled water.

For 2-D gel analysis, BN-gels were excised into strips after running. The gel strips were soaked in the sodium dodecyl sulfate (SDS) sample buffer (62.5 mM Tris-Cl pH 8.5, 2% (w/v) SDS, 5% (w/v) β-mercaptoethanol, 10% (w/v) glycerol and a trace of bromophenol blue in the presence of 50 mM dithiothreitol (DTT) for 15 min at room temperature and then soaked in the SDS sample buffer containing 20% (v/v) ethanol in the presence of 5 mM DTT for 15 min at room temperature. The strips were applied on 5-20% gradient SDS-polyacrylamide gel electrophoresis (SDS-PAGE) with overlaying melted 0.5% (w/v) agarose in the SDS sample buffer to attach the gel strips onto the gel.

#### Western blotting

Proteins separated by 12.5% or 10% SDS-PAGE or BN-PAGE were transferred to immobilon-P (PVDF) membranes (Millipore) using semi-dry blotting systems. For BN-PAGE, the PDVF membranes were washed with 8% (v/v) acetic acid for 15 min to fix the proteins on the membrane and washed twice with distilled water. After blocking with 5% of skim milk in TTBS (20 mM Tris-Cl pH 7.5, 137 mM NaCl and 0.1% (v/v) Tween20), the membranes were reacted with the proper dilutions of the primary antibodies in TTBS containing 3% (w/v) of bovine serum albumin and 0.05% (w/v) of sodium azide for 1 hour. After 2 times of washing with TTBS for 5 min each, the membranes were reacted with secondary antibodies, goat anti-rabbit IgG-HRP (horseradish peroxidase) or goat anti-mouse IgG-HRP (Invitrogen) at dilution 1:10,000, for 40 min. The membranes were washed 4 times with TTBS for 10 min each and developed using the Western Lightning Chemiluminescence Reagent (PerkinElmer) according to the manufacture’s instruction.

#### Immunofluorescence microscopy

*L. pneumophila* cells grown in AYE medium (1 ml) were harvested by microcentrifugation. The pellet was suspended with 1 ml of 20 mM cold HEPES pH 7.4 and fixed with final 4% (w/v) of Paraformaldehyde for 1 hour on ice. The fixed cells were washed with phosphate-buffered saline (PBS) and resuspended with 1 ml of PBS containing 0.05% (w/v) of sodium azide. Fixed bacterial cells (10 ul) were spun down (1000 rpm, 5 min) onto a coverslip placed in a 24-well dish containing lysozyme buffer (2.5 mM Tris-Cl pH 8.0, 1.8% (w/v) glucose and 10 mM EDTA). Cells were treated with final 2 mg/ml lysozyme at 37 °C for 30 minutes to permeabilize bacterial peptideglycan layer. The bacteria-mounted coverslips were soaked in methanol at −20 °C for 10 seconds to permeabilize bacterial membranes and gently washed with PBS. After blocking with 2% (w/v) goat serum in PBS for 30 min at 37 °C, proteins were visualized by incubation with anti-M45 or anti-DotF antibodies, followed by rhodamine red-X goat anti-rabbit IgG (Life Technologies, R6393). Digital images were acquired with a Nikon TE2000-U microscope using a x100 objective lens and Hamamatsu ORCA camera controlled by NIS-Elements software.

#### Size exclusion chromatography with multi-angle light scattering (SEC-MALS)

SEC-MALS measurements were conducted using a TOSOH 8020 HPLC system (TOSOH) equipped with a Dawn EOS 18-angle light scattering detector with an Optilab rEX online refractive index detector (Wyatt). The absolute molecular mass was calculated by analyzing the scattering data using Wyatt’s ASTRA analysis software (Wyatt Technology). Protein samples were separated on a Superdex S-200 10/300 analytical gel filtration column (GE Healthcare) with flow-rate of 0.4 ml/min. Bovine serum albumin (BSA) was used for calibration.

A 0.1 ml of full-length TraM at a concentration of 1 mg/ml was injected and eluted in 20 mM Tris-HCl pH 7.5, 300 mM NaCl, 0.05% DDM. The molecular masses of TraM and DDM were determined by the dual detection method implemented in the conjugated analysis mode of the ASTRA analysis software. The refractive index increments of protein and DDM used were 0.185 ml g^−1^ and 0.133 ml g^−1^, respectively. The extinction coefficient of TraM for UV detection at 280 nm was calculated from the amino acid sequence.

DotI_C_ was incubated, loaded and eluted in 100 mM CAPS-NaOH pH 11.0, 200 mM NaCl, 20% MPD. The absolute molecular mass was calculated using Zimm’s model implemented in the ASTRA analysis software.

#### Crystallization

Protein purification procedures are described in supporting material and methods. Crystal screening was performed by the sitting-drop vapour-diffusion technique with commercially available screening kits Wizard I and II (Emerald BioSystems) and Crystal Screen I and II (Hampton Research) at 4 °C and 20 °C. Each drop was prepared by mixing 0.5 μl of protein solution with 0.5 μl of reservoir solution, and equilibrated to 70 μl of reservoir solution. DotI_C_ crystals were grown from various conditions containing low-molecular-weight polyethylene glycol (PEG) or MPD as a precipitant under neutral to alkaline pH. After optimization of the condition, we obtained rod-shape crystals (from I) and cubic-shape crystals (form II). The form I crystals were grown from the drops prepared by mixing 0.5 μl of DotI_C_ solution (5 mg/ml) with 0.5 μl of reservoir solution containing 40% (v/v) MPD and 0.1 M CAPS-NaOH pH10.5 at 4 °C by the sitting-drop method. The space group of the crystals was orthorhombic *P*2_1_2_1_2 with unit cell dimensions a = 145.6, b = 207.6, and c = 58.2 Å. The form II crystals were grown from the mixture of 0.5 μl of DotI_C_ solution (5 mg/ml) and 0.5 μl of reservoir solution containing 40% (v/v) PEG300, 0.2 M NaCl and 0.1 M HEPES-NaOH pH7.5 at 4 °C by the sitting-drop method. The space group of the form II crystals was cubic *I*432 with unit cell dimensions a = b = c = 230.3 Å.

TraM_C_ crystals were grown from drops containing Li_2_SO_4_ or NaCl under neutral pH at 20 °C. The crystals suitable for X-ray experiment were obtained by mixing 1.5 μl of TraM_C_ solution (12.5 mg/ml) with 1.5 μl of reservoir solution containing 0.8 M Li_2_SO_4_ and 0.05 M glycine-NaOH pH 9 at 20°C by the hanging-drop method. The space group of the crystals was *P*4_3_2_1_2 with unit cell dimensions a = b = 67.8 and c = 73.6 Å.

Se-Met derivative crystals of DotI_C_ and TraM_C_ were grown from the same conditions as for their native crystals.

#### Data collection and structural determination

X-ray experiment was carried out at beamlines BL32XU and BL41XU in SPring-8 (Harima, Japan) with the approval of the Japan Synchrotron Radiation Research Institute (JASRI) (Proposal No. 2010B1901 and 2011A1240). DotI_C_ Crystals were frozen by directly plunging them into liquid nitrogen because the crystallization drop contains about 40% (v/v) MPD or PEG300, which are enough for cryo-protection. The diffraction data recorded on a MX225HE CCD detector (Rayonix) under nitrogen gas flow (−173 °C) were processed with MOSFLM^30^ and scaled with SCALA^31^. The initial experimental phase of the DotI_C_ form I crystal was calculated using the SAD data of the Se-Met derivative with a program Phenix^32^. The atomic model of DotI_C_ was constructed with Coot^33^ and refined with Phenix using the Se-Met derivative data which showed the highest resolution of 2.2 Å. The refinement R factor and the free R factor were converged to 19.1% and 23.5%, respectively. The Ramachandran plot indicated that 92.7% and 7.3% residues were located in the most favorable and allowed region, respectively. The DotI_C_ form II crystal structure was solved by the molecular replacement method with Phenix using a subunit structure of the form I crystal. The model was modified with Coot and refined to 3.5 Å resolution with Phenix. The final refinement R and the free R factors were 17.9% and 21.6%, respectively, and 86.6% and 13.4% residues were located in the most favorable and allowed region in the Ramachandran plot, respectively.

TraM_C_ crystals were soaked in a cryo-protectant solution containing 10% (v/v) glycerol and 90% (v/v) of the reservoir solution for a few seconds, and were immediately frozen in liquid nitrogen. The diffraction data were corrected, processed and scaled in the same way as DotI_C_. The structure of TraM_C_ was also determined by SAD method using the anomalous data of Se-Met derivative with Phenix. The model was built with Coot and refined to 1.5 Å resolution with Phinix. The final refinement R factor and the free R factor were 18.0% and 20.7%, respectively. The Ramachandran plot indicated that 97.5% and 2.5% residues were located in the most favorable and allowed region, respectively. The statistics of diffraction data and structural refinement are summarized in Table S2.

The atomic coordinates have been deposited in Protein Data Bank, www.pdb.org (PDB ID code 3WZ3, 3WZ4 and 3WZ5).

## Additional Information

**How to cite this article**: Kuroda, T. *et al.* Molecular and structural analysis of *Legionella* DotI gives insights into an inner membrane complex essential for type IV secretion. *Sci. Rep.* **5**, 10912; doi: 10.1038/srep10912 (2015).

## Supplementary Material

Supplementary Information

## References

[b1] Alvarez-Martinez C. E. & Christie P. J. Biological diversity of prokaryotic type IV secretion systems. Microbiol Mol Biol Rev 73, 775–808 (2009). 19946141 10.1128/MMBR.00023-09 PMC2786583

[b2] Christie P. J., Atmakuri K., Krishnamoorthy V., Jakubowski S. & Cascales E. Biogenesis, architecture, and function of bacterial type IV secretion systems. Annu Rev Microbiol 59, 451–485 (2005). 16153176 10.1146/annurev.micro.58.030603.123630 PMC3872966

[b3] Fronzes R., Christie P. J. & Waksman G. The structural biology of type IV secretion systems. Nat Rev Microbiol 7, 703–714 (2009). 19756009 10.1038/nrmicro2218 PMC3869563

[b4] Christie P. J. & Vogel J. P. Bacterial type IV secretion: conjugation systems adapted to deliver effector molecules to host cells. Trends Microbiol 8, 354–360. (2000). 10920394 10.1016/s0966-842x(00)01792-3 PMC4847720

[b5] Chandran V. *et al.* Structure of the outer membrane complex of a type IV secretion system. Nature 462, 1011–1015 (2009). 19946264 10.1038/nature08588 PMC2797999

[b6] Fronzes R. *et al.* Structure of a type IV secretion system core complex. Science 323, 266–268 (2009). 19131631 10.1126/science.1166101 PMC6710095

[b7] Rivera-Calzada A. *et al.* Structure of a bacterial type IV secretion core complex at subnanometre resolution. Embo J 32, 1195–1204 (2013). 23511972 10.1038/emboj.2013.58 PMC3630358

[b8] Low H. H. *et al.* Structure of a type IV secretion system. Nature 508, 550–553 (2014). 24670658 10.1038/nature13081 PMC3998870

[b9] Komano T., Yoshida T., Narahara K. & Furuya N. The transfer region of IncI1 plasmid R64: similarities between R64 tra and *Legionella icm/dot* genes. Mol Microbiol 35, 1348–1359 (2000). 10760136 10.1046/j.1365-2958.2000.01769.x

[b10] Wilkins B. M. & Thomas A. T. DNA-independent transport of plasmid primase protein between bacteria by the I1 conjugation system. Mol Microbiol 38, 650–657 (2000). 11069687 10.1046/j.1365-2958.2000.02164.x

[b11] Nagai H. & Kubori T. Type IVB Secretion Systems of *Legionella* and Other Gram-Negative Bacteria. Frontiers in microbiology 2, 136 (2011). 21743810 10.3389/fmicb.2011.00136 PMC3127085

[b12] Kubori T. *et al.* Native structure of a type IV secretion system core complex essential for *Legionella* pathogenesis. Proc Natl Acad Sci U S A 111, 11804–11809 (2014). 25062693 10.1073/pnas.1404506111 PMC4136560

[b13] Andrews H. L., Vogel J. P. & Isberg R. R. Identification of linked *Legionella pneumophila* genes essential for intracellular growth and evasion of the endocytic pathway. Infect Immun 66, 950–958 (1998). 9488381 10.1128/iai.66.3.950-958.1998 PMC108001

[b14] Berger K. H. & Isberg R. R. Two distinct defects in intracellular growth complemented by a single genetic locus in *Legionella pneumophila*. Mol Microbiol 7, 7–19 (1993). 8382332 10.1111/j.1365-2958.1993.tb01092.x

[b15] Segal G., Purcell M. & Shuman H. A. Host cell killing and bacterial conjugation require overlapping sets of genes within a 22-kb region of the *Legionella pneumophila* genome. Proc Natl Acad Sci USA 95, 1669–1674 (1998). 9465074 10.1073/pnas.95.4.1669 PMC19142

[b16] Segal G. & Shuman H. A. *Legionella pneumophila* utilizes the same genes to multiply within *Acanthamoeba castellanii* and human macrophages. Infect Immun 67, 2117–2124 (1999). 10225863 10.1128/iai.67.5.2117-2124.1999 PMC115946

[b17] Vincent C. D. *et al.* Identification of the core transmembrane complex of the *Legionella* Dot/Icm type IV secretion system. Mol Microbiol 62, 1278–1291 (2006). 17040490 10.1111/j.1365-2958.2006.05446.x

[b18] Holm L., Kaariainen S., Rosenstrom P. & Schenkel A. Searching protein structure databases with DaliLite v.3. Bioinformatics 24, 2780–2781 (2008). 18818215 10.1093/bioinformatics/btn507 PMC2639270

[b19] Bailey S., Ward D., Middleton R., Grossmann J. G. & Zambryski P. C. *Agrobacterium tumefaciens* VirB8 structure reveals potential protein-protein interaction sites. Proc Natl Acad Sci U S A 103, 2582–2587 (2006). 16481621 10.1073/pnas.0511216103 PMC1413848

[b20] Terradot L. *et al.* Structures of two core subunits of the bacterial type IV secretion system, VirB8 from *Brucella suis* and ComB10 from *Helicobacter pylori*. Proc Natl Acad Sci U S A 102, 4596–4601 (2005). 15764702 10.1073/pnas.0408927102 PMC555499

[b21] Goessweiner-Mohr N. *et al.* The 2.5 A structure of the *Enterococcus* conjugation protein TraM resembles VirB8 type IV secretion proteins. J Biol Chem 288, 2018–2028 (2013). 23188825 10.1074/jbc.M112.428847 PMC3548508

[b22] Porter C. J. *et al.* The conjugation protein TcpC from *Clostridium perfringens* is structurally related to the type IV secretion system protein VirB8 from Gram-negative bacteria. Mol Microbiol 83, 275–288 (2012). 22150951 10.1111/j.1365-2958.2011.07930.x

[b23] Nagai H. & Roy C. R. The DotA protein from *Legionella pneumophila* is secreted by a novel process that requires the Dot/Icm transporter. EMBO J. 20, 5962–5970 (2001). 11689436 10.1093/emboj/20.21.5962 PMC125688

[b24] Judd P. K., Kumar R. B. & Das A. Spatial location and requirements for the assembly of the *Agrobacterium tumefaciens* type IV secretion apparatus. Proc Natl Acad Sci U S A 102, 11498–11503 (2005). 16076948 10.1073/pnas.0505290102 PMC1183602

[b25] Aguilar J., Zupan J., Cameron T. A. & Zambryski P. C. *Agrobacterium* type IV secretion system and its substrates form helical arrays around the circumference of virulence-induced cells. Proc Natl Acad Sci U S A 107, 3758–3763 (2010). 20133577 10.1073/pnas.0914940107 PMC2840527

[b26] Cascales E. & Christie P. J. Definition of a bacterial type IV secretion pathway for a DNA substrate. Science 304, 1170–1173 (2004). 15155952 10.1126/science.1095211 PMC3882297

[b27] Feeley J. C. *et al.* Charcoal-yeast extract agar: primary isolation medium for *Legionella pneumophila*. J Clin Microbiol 10, 437–441 (1979). 393713 10.1128/jcm.10.4.437-441.1979 PMC273193

[b28] Nakano N., Kubori T., Kinoshita M., Imada K. & Nagai H. *Crystal Structure of* Legionella *DotD: insights into the relationship between type IVB and type II/III secretion systems*. PLoS Pathog 6, e1001129 (2010). 20949065 10.1371/journal.ppat.1001129 PMC2951367

[b29] Roy C. R., Berger K. & Isberg R. R. *Legionella pneumophila* DotA protein is required for early phagosome trafficking decisions that occur within minutes of bacterial uptake. Mol Microbiol 28, 663–674 (1998). 9632267 10.1046/j.1365-2958.1998.00841.x

[b30] Leslie A. G. W. CCP4+ESF-EACMB. Newslett Protein Crystallogr 26, 27–33 (1992).

[b31] Winn M. D. *et al.* Overview of the CCP4 suite and current developments. Acta Crystallogr D Biol Crystallogr 67, 235–242 (2011). 21460441 10.1107/S0907444910045749 PMC3069738

[b32] Adams P. D. *et al.* PHENIX: building new software for automated crystallographic structure determination. Acta Crystallogr D Biol Crystallogr 58, 1948–1954 (2002). 12393927 10.1107/s0907444902016657

[b33] Emsley P. & Cowtan K. Coot: model-building tools for molecular graphics. Acta Crystallogr D Biol Crystallogr 60, 2126–2132 (2004). 15572765 10.1107/S0907444904019158

