# Supplementary material for: Molecular and structural analysis of Legionella DotI gives insights into an inner membrane complex essential for type IV secretion
Source: Sci Rep. 2015 Jun 3;5:10912. doi: 10.1038/srep10912 (PMC4454188; doi:10.1038/srep10912)
Supplement: Supplementary Information [file srep10912-s1.pdf]

Supplementary Information

Molecular and structural analysis of *Legionella* DotI gives insights into an inner membrane complex essential for type IV secretion.

Takuya Kuroda, Tomoko Kubori, Xuan Thanh Bui, Akihiro Hyakutake, Yumiko Uchida, Katsumi Imada and Hiroki Nagai

Supplementary Methods

Bacterial strains and construction of plasmids.

L. pneumophila strains with *dot/icm* gene deletions were constructed by allelic exchange [1]. The luminescent CR39*luc* and CR419*luc* strains were obtained by inserting the *P. luminescence lux* operon into the *L. pneumophila* chromosomes of CR39 and CR419, respectively [2]. For expression in *Legionella*, M45-tagged or un-tagged clones were constructed using PCR-amplification and cloning into pMMB207NT [3] or pMMB207 [4], respectively. The Strep-tag and Strep-3xFLAG tag cloning vectors based on pMMB207 or on pET15b were constructed as indicated in the Table S1. Introduction of the alanine-substitution mutations of *dotI* was conducted by site-directed mutagenesis using QuickChange II Site-Directed Mutagenesis Kit (Agilent Technologies) according to the manufacturer's instructions.

Purification of DotI-DotJ complex. Lemo21 (DE3) carrying pstrep-3xFLAG-*dotJ dotI* (pNH1617) was grown to logarithmic phase at 37°C in 1L L-broth. After addition of final 0.4 mM IPTG to induce the production of DotI-DotJ complex, cells were cultured for additional 6 hours at 25°C. *E. coli* cells were recovered by centrifugation, and suspended with buffer A (20mM Tris HCl pH 7.5, 300mM NaCl) containing Complete protease inhibitor cocktail (Roche). After adding final 10 µg/ml DNase I, cells were disrupted by two passages of French pressure cell. Lysate was centrifuged (3,000 g, 15 min) to remove undisrupted cells, and final 5 mM EDTA was added to the supernatant (whole cell lysate). Crude membranes was collected by ultracentrifuation at 100,000 g for one hour (Type70Ti), and suspended with 10 ml of buffer B (20mM Tris HCl pH 7.5, 300mM NaCl, 1mM EDTA). To crude membranes, final 1% (w/v) DDM was added to solubilize the DotI-DotJ complex and incubated for two hours at 4°C with rotation. Insoluble material was removed by ultracentrifugation as above, and

the complex was pulled down using Streptacin sepharose (GE healthcare). Resins were washed three times with buffer C (20mM Tris HCl pH 7.5, 300mM NaCl, 1mM EDTA, 0.1% (w/v) DDM), and the DotI-DotJ complexes were eluted with 2.5mM desthiobiotin (Sigma-Aldrich) in buffer C.

Purification of R64 TraM complex. TraM complex was purified essentially as described for purification of the DotI-DotJ complex with a couple of modifications: BL21(DE3) pET15b-$TraM_{FL}$ (pNH1609) was employed as host *E. coli* and affinity purification was carried out using HisSelect resin (Sigma-Aldrich). Resins were washed with 30mM imidazole in buffer C and the TraM complex was eluted with 100mM imidazole in buffer C.

Purification of $DotI_C$ and $TraM_C$ for crystallization. N-terminally His6-tagged $DotI_C$ (residues 73-212 of DotI) was overproduced in *E. coli* BL21(DE3) from the plasmid pNH1359 and purified. The frozen stock cells were thawed and suspended into 80 ml of buffer (50 mM Tris-HCl pH7.5, 1 mM EDTA) with 2 tablets of protease inhibitor cocktail Complete EDTA free and 22 mg of lysozyme. After incubation at 4°C for 40 min, the cells were sonicated (ASTRASON), and the cell lysate was centrifuged (18,000 g x 15 min at 4°C) to remove cell debris. The supernatant was loaded on a SP-sepharose column (GE Healthcare) equilibrated with 20 mM Tirs-HCl pH8.0. Proteins were eluted with a linear gradient of 0 - 2 M NaCl. The fractions containing His-$DotI_C$ were loaded on a HiTrap chelating column (GE Healthcare) equilibrated with binding buffer (20 mM Tris-HCl pH7.0, 600 mM NaCl) and eluted using a linear gradient of 1 - 500 mM imidazole. The His-tag was then removed using proteolytic cleavage by adding thrombin to the His-$DotI_C$ solution and dialyzed overnight against binding buffer at 4°C. The reactant was purified on a HiTrap Benzamidine FF column (GE Healthcare) followed by the HiTrap Chelating column to remove thrombin and non-cleaved His-$DotI_C$. The $DotI_C$

solution was loaded on a HiLoad Superdex 75 (26/60) column (GE Healthcare), and eluted with the binding buffer. The peak fractions were pooled and concentrated for further use. The purity of the product was examined by SDS-PAGE and MALDI-TOF mass spectrometry (Voyager DE/PRO). Se-Met labeled proteins were purified in the same way as for native proteins.

N-terminally His6-tagged $TraM_C$ (residues 91-230) was overproduced in *E. coli* BL21(DE3) from the plasmid pNH1377 and purified as described for $DotI_C$ with a few modifications. Buffers at pH 7.0 were employed for all preparation steps. Cleared lysate was subjected to Q-sepharose column (GE Healthcare) and flow through fraction containing His-$TraM_C$ was subjected to affinity purification using HisSelect resins (Sigma-Aldrich). The His-$TraM_C$-bound resins were washed with buffer containing 10 mM imidazole and His-$TraM_C$ was eluted with buffer containing 100 mM imidazole. Thrombin His-tag was removed by incubation with thrombin (10 U/mg protein) at 22°C for 30 minutes. Cleaved His-tag and thrombin were removed by passing through benzamidine-sepharose (GE Healthcare) and HisSelect columns.

Intracellular growth assay.

U937 human macrophage-like cells were grown in RPMI 1640 media (Gibco 11875) containing 10% fetal bovine serum (FBS). Semi-confluent cells were treated with 100 ng/ml of phorbol myristate acetate (PMA, Sigma P1585) to differentiate to monocytes for 48 hours. The cells were related into the 24-well tissue culture dishes (Falcon) and were infected with *L. pneumophila* strains at a multiplicity of infection (MOI) of 2. Uninfected bacteria were removed by washing the cells with 1 ml of DPBS (37°C, Gibco) twice after 1 hour of infection and the cells were incubated in 0.5 ml of pre-warmed (37°C) fresh media containing the serum. The cells were lysed with 1 ml of sterile water at 1, 24, 48

and 72 hours after infection. Serial dilutions of the lysates were plated on CYE containing 12.5 μg/ml of chloramphenicol to measure colony-forming units (CFUs).

L. pneumophila growth assay of luminescent *L. pneumophila* in *Acanthamoeba castellanii* was conducted as previously described [2] with the following modifications. *A. castellanii* was grown in 25 ml of peptone – yeast extract – glucose media (PYG) [5] in 75 cm^2 tissue culture flasks at 25°C without aeration to confluence (2-3 days). Before an infection experiment, *A. castellanii* cells were washed 2 times with *A. castellanii* buffer (Ac buffer; PYG media without peptone – yeast extract – glucose) and seeded onto a 96-well format plate (maker) at 10^5 cells per well in a total volume of 200 µl for 3 hours to let the amoebae adhere. *L. pneumophila* strains expressing luciferase was added to the wells at a MOI of 2. The infected amoeba was incubated at 37°C, and luminescence was measured at indicated time points using the luminescence microplate reader (Powerscan HT, BioTek).

References cited in supplementary information

1 Zuckman, D. M., Hung, J. B. & Roy, C. R. Pore-forming activity is not sufficient for *Legionella pneumophila* phagosome trafficking and intracellular growth. *Mol Microbiol* **32**, 990-1001 (1999).

2 Coers, J., Vance, R. E., Fontana, M. F. & Dietrich, W. F. Restriction of *Legionella pneumophila* growth in macrophages requires the concerted action of cytokine and Naip5/Ipaf signalling pathways. *Cell Microbiol* **9**, 2344-2357 (2007).

3 Coers, J. *et al.* Identification of *icm* protein complexes that play distinct roles in the biogenesis of an organelle permissive for *Legionella pneumophila* intracellular growth. *Mol Microbiol* **38**, 719-736. (2000).

4 Morales, V. M., Backman, A. & Bagdasarian, M. A series of wide-host-range low-copy-number vectors that allow direct screening for recombinants. *Gene* **97**, 39-47 (1991).

5 Moffat, J. F. & Tompkins, L. S. A quantitative model of intracellular growth of *Legionella pneumophila* in *Acanthamoeba castellanii*. *Infect Immun* **60**, 296-301 (1992).

6 Studier, F. W. & Moffatt, B. A. Use of bacteriophage T7 RNA polymerase to direct selective high-level expression of cloned genes. *J Mol Biol* **189**, 113-130 (1986).

7 Berger, K. H. & Isberg, R. R. Two distinct defects in intracellular growth complemented by a single genetic locus in *Legionella pneumophila*. *Mol Microbiol* **7**, 7-19 (1993).

8 Ninio, S., Zuckman-Cholon, D. M., Cambronne, E. D. & Roy, C. R. The *Legionella* IcmS-IcmW protein complex is important for Dot/Icm-mediated protein translocation. *Mol Microbiol* **55**, 912-926 (2005).

9 Nakano, N., Kubori, T., Kinoshita, M., Imada, K. & Nagai, H. Crystal Structure of *Legionella* DotD: insights into the relationship between type IVB and type II/III secretion systems. *PLoS Pathog* **6**, e1001129 (2010).

10 Kubori, T. *et al.* Native structure of a type IV secretion system core complex essential for *Legionella* pathogenesis. *Proc Natl Acad Sci U S A* (2014).

Table S1. Bacterial strains and plasmids used in this study.

Strain or plasmid	genotype	Reference
**Strains**		
Escherichia coli		
BL21 (DE3)	F^-*ompT* *hsd*S_B (r_B^-, m_B^-) *gal dcm* (DE3)	[6]
DH5αλpir	DH5α(λpir) *tet*::Mu	[6]
Lemo21 (DE3)	fhuA2 [lon] ompT gal (λDE3) [dcm] ΔhsdS/ pLemo (CamR)	New England Biolabs
L. pneumophila		
CR39	*L. pneumophila* serogroup1, strain Lp01 *rpsL*	[7]
CR416	Lp01 Δ*icmX*	[8]
CR157	Lp01 Δ*icmW*	[1]
NH1005	Lp01 Δ*icmV*	This study
CR276	Lp01 Δ*dotA*	Roy C. (unpublished)
NH1015	Lp01 Δ*dotB*	This study
NH1016	Lp01 Δ*dotC*	[9]
NH1020	Lp01 Δ*dotD*	[9]
NH1031	Lp01 Δ*dotK*	This study
NH1033	Lp01 Δ*dotJ*	This study
CR419	Lp01 Δ*dotI*	[8]
NH1021	Lp01 Δ*dotH*	[9]
NH1022	Lp01 Δ*dotG*	[9]
NH1055	Lp01 Δ*dotF*	[9]
NH1024	Lp01 Δ*dotE*	This study
NH1035	Lp01 Δ*dotP*	This study
NH1037	Lp01 Δ*dotO*	This study
NH1075	Lp01 Δ*icmXWV*Δ*dotA*Δ*dotBCD*Δ*icmT-dotU*	[10]
NH1205	Lp01 Δ*dotJ* Δ*dotI*	This study
CR39luc	CR39 expressing luciferase	This study
CR419luc	CR419 expressing luciferase	This study

Plasmids

pET15b		
	NovagenpMMB207	cloning
vector derived from RSF1010		[4]
pMMB207NT	Amino-terminal M45 epitope tag vector derived from pMMB207	[3]
pNH1526	Amino-terminal Strep tag vector derived from pMMB207	This study
pNH1582	Amino-terminal Strep-3xFLAG tag vector derived from pMMB207	This study
pNH1618	Amino-terminal Strep-thrombin cleavage site vector from pET15b	This study
pNH1593	pMMB207 encoding Strep-3xFLAG-*dotJ dotI*	This study
pNH1359	pET15b encoding His-*dotI*$_{73\text{-}212}$	This study
pNH1617	pET15b encoding Strep-3xFLAG-*dotJ dotI*	This study
pNH1619	pET15b encoding Strep-thrombin cleavage site-*dotJ dotI*	This study
pNH1609	pET15b encoding His-*traM*$_{FL}$	This study
pNH1377	pET15b encoding His-*traM*$_{91\text{-}224}$	This study
pNH1265	pMMB207NT encoding M45-*dotG*	This study
pNH1548	pMMB207NT encoding M45-*dotI*	This study
pNH1577	pMMB207NT encoding M45-*dotJ*	This study
pNH1583	pMMB207NT encoding M45-*dotJ dotI*	This study
pNH1594	pMMB207NT encoding M45-*dotI*$_{D75A}$	This study
pNH1595	pMMB207NT encoding M45-*dotI*$_{Y97A}$	This study
pNH1623	pMMB207NT encoding M45-*dotI*$_{R98A}$	This study
pNH1624	pMMB207NT encoding M45-*dotI*$_{Q102A}$	This study
pNH1625	pMMB207NT encoding M45-*dotI*$_{W113A}$	This study
pNH1597	pMMB207NT encoding M45-*dotI*$_{D114A}$	This study
pNH1598	pMMB207NT encoding M45-*dotI*$_{R142A}$	This study
pNH1599	pMMB207NT encoding M45-*dotI*$_{Q148A}$	This study
pNH1630	pMMB207NT encoding M45-*dotI*$_{R98A\ Q102A}$	This study

Construction of pNH1526 (Amino-terminal Strep tag vector derived from pMMB207)

A region of pMMB207NT was PCR-amplified using a primer inside *lacI* gene with MluI site (5’-ctgacgcgttgcgcgagaag-3’) and a primer containing strep-tag sequence with BamHI site (5’-gcggatccgggcgccTTTTTCGAACTGCGGGTGGCTCCAgctagccattattaccactcctgagctaaatctcttac-3

’). The amplified fragment was cloned into pMMB207 using *Mlu*I/*Bam*HI sites, resulting in replacement of the M45 tag with Strep tag.

Construction of pNH1582 (Amino-terminal Strep-3xFLAG tag vector derived from pMMB207)

Annealed oligonucleotides having 3xFLAG sequence having GATC overhangs at the ends (5’-gatcatggactacaaagaccatgacggtgattataaagatcatgacatcgattacaaggatgacgatgacaagcg-3’; 5’-gatccgcttgtcatcgtcatccttgtaatcgatgtcatgatctttataatcaccgctatggtctttgtagtccat-3’) was cloned into *Bam*HI sites of pNH1526.

Construction of pNH1618 (Amino-terminal Strep-thrombin cleavage site vector from pET15b)

A region of pET15b was PCR-amplified using a primer containing NcoI-Strep-thrombin cleavage sequence (5’-cgcgccATGgctagcTGGAGCCACCCGCAGTTCGAAAAAagcggcCTGGTGCCGCGCGGCAGC-3’) and a primer containing PstI site (5’-TTGCTGCAGG CATCGTGGTG TCAC-3’). The amplified fragment was cloned into pET15b using NcoI/PstI sites.

Construction of pNH1593 (pMMB207 encoding Strep-3xFLAG-*dotJ dotI*)

PCR-amplified *dotJ*-*dotI* locus having *Bgl*II and *Xba*I sites was cloned into pNH1582 using BamHI/XbaI sites.

Construction of pNH1359 (pET15b encoding His-*dotI*$_{73\text{-}212)}$

PCR-amplified *dotI*$_{C}$ (73-212aa) locus was cloned into pET15b using *Nde*I/*Bam*HI sites.**Construction of pNH1617 (pET15b encoding Strep-3xFLAG-*dotJ dotI)***

PCR-amplified strep-3xFLAG-*dotJ dotI* locus on pNH1593 using a primer having NcoI site and N-terminal portion of the Strep tag sequence (5’-tataccatggctagcTGGAGCCAC-3’) and a primer having XhoI site (5’-gcctcgagctaggataccccaccgctggc-3’).
was cloned into pET15b using *Nco*I/*Xho*I sites.

Construction of pNH1619 (pET15b encoding Strep-thrombin cleavage site- *dotJ dotI)*

PCR-amplified *dotJ dotI* locus was cloned into pNH1618 using XhoI site.

Construction of pNH1609 (pET15b encoding His-*traM*$_{FL}$)

PCR-amplified fragments from R64 plasmid were cloned into pET15b using *Nde*I/*Bam*HI sites.

Construction of pNH1377 (pET15b encoding His-*traM*$_{91\text{-}224}$)

PCR-amplified fragments from R64 plasmid were cloned into pET15b using *Nde*I/*Bam*HI sites.

Table S2. Data collection and X-ray refinement statistics

	DotI$_C$ form I	DotI$_C$ form II	TraM$_C$
Space group	$P2_12_12$	$I432$	$P4_32_12$
Cell dimensions			
a, b, c (Å)	145.6, 207.6, 58.2	230.3, 230.3, 230.3	67.8, 67.8, 73.6
Wavelength (Å)	0.97923	0.97923	0.97910
Resolution (Å)	54.0-2.2 (2.32-2.2)	81.4-3.5 (3.69-3.5)	24.9-1.5 (1.58-1.5)
R_{merge}	12.7 (32.1)	12.5 (24.2)	11.5 (35.1)
Mean(I/sd(I))	9.3 (4.0)	16.7 (10.4)	17.4 (8.9)
Completeness (%)	98.4 (94.6)	100 (100)	99.0 (98.0)
Anomalous (%)	92.5 (78.9)	100 (100)	99.2 (98.2)
Redundancy	5.8 (4.5)	11.5 (11.8)	21.1 (21.4)
Anomalous	3.1 (2.6)	5.9 (5.9)	11.3 (11.2)
Resolution range (Å)	37.4-2.2 (2.23-2.2)	72.8-3.5 (3.77-3.5)	24.0-1.5 (1.54-1.5)
Number of reflections			
working set	88,906 (5,788)	13,465(2,516)	27,723 (1,765)
test set	2,000 (133)	665 (115)	2,008 (144)
R_w (%)	19.1 (21.1)	17.9 (18.9)	18.0 (18.0)
R_{free} (%)	23.5 (26.8)	21.6 (25.1)	20.7 (21.5)
Rms deviation			
bond length (Å)	0.003	0.004	0.009
bond angle (°)	0.63	0.74	1.22
B-factors			
Protein atoms	19.8	43.0	12.3
Ligand atoms	27.6	-	14.8
Solvent atoms	25.8	-	26.6
Ramachandran plot (%)			
Most favored	92.7	86.6	97.5
Additionally allowed	7.3	13.4	2.5
Generously allowed	0	0	0
Disallowed	0	0	0
Number of atoms			
protein	8,578	4,196	1,104
ligand	256	0	5
solvent	1,241	0	230

Values in parentheses are for the highest resolution shell.

$R_w = \Sigma \| Fo | - | Fc \| / \Sigma | Fo |$, $R_{free} = \Sigma \| Fo | - | Fc \| / \Sigma | Fo |$

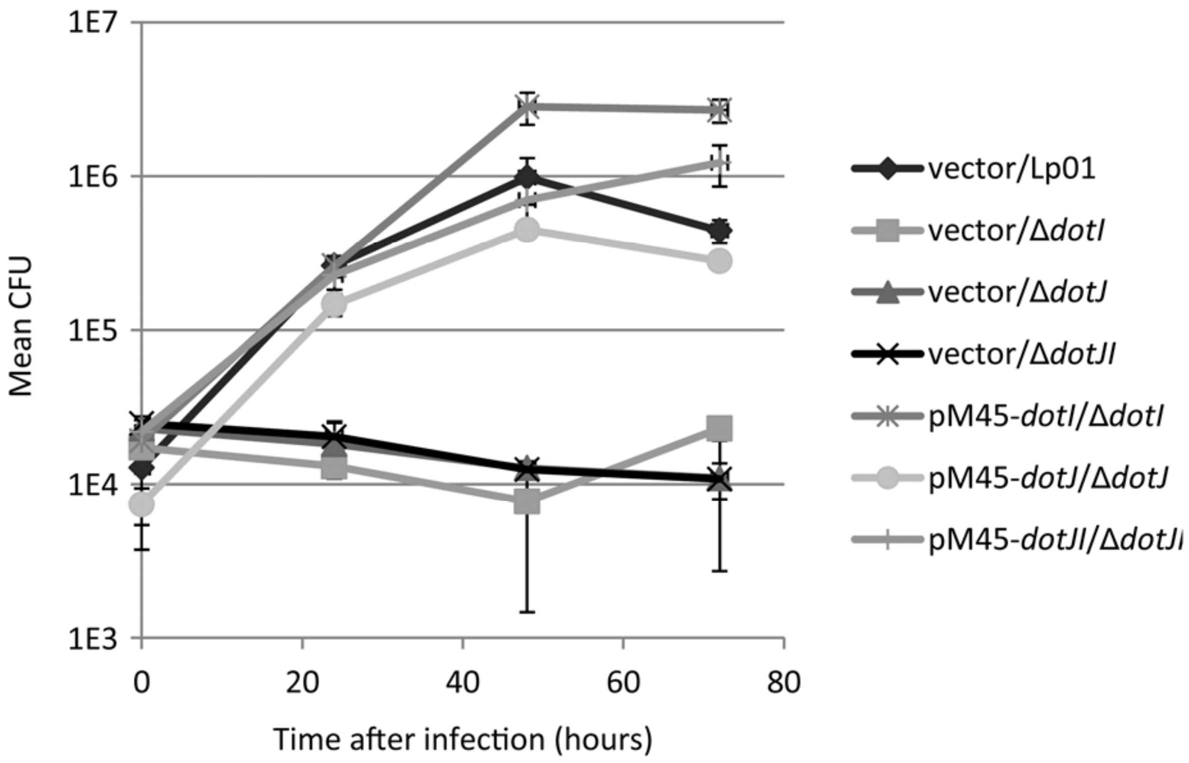

Fig. S1. Intracellular growth assay of *dotI/dotJ* deletion strains. U937 macrophage-like cells were infected with the indicated *L. pneumophila* strains (MOI 2). CFUs were counted at the indicated time points.

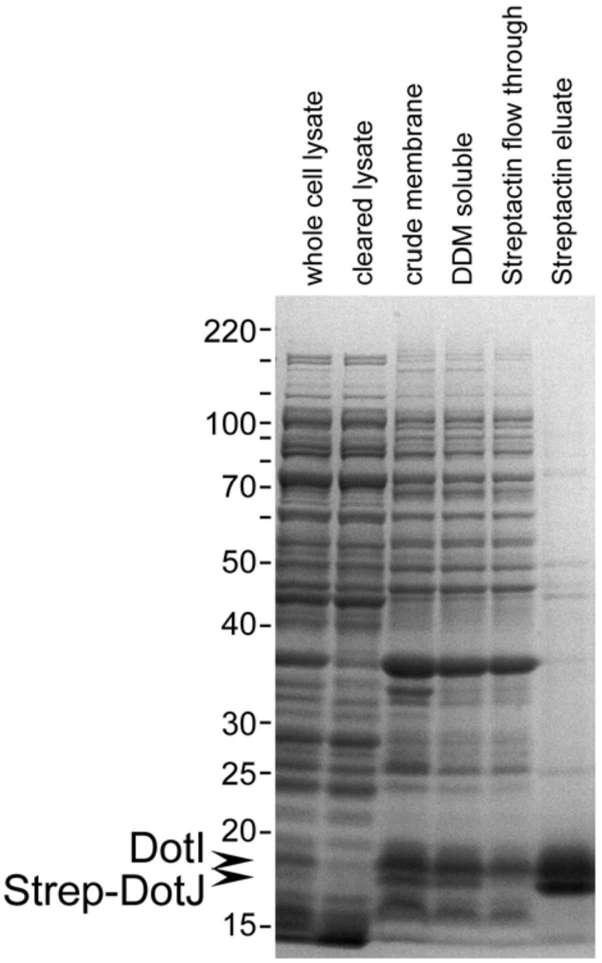

Fig. S2. Purification of DotI-DotJ complex. The complex was purified from *E. coli* producing full length DotI and Strep-DotJ as described in Material and Methods. Indicated fractions were subjected to 12.5% SDS PAGE analysis and proteins were stained by Coomassie Brilliant Blue staining.

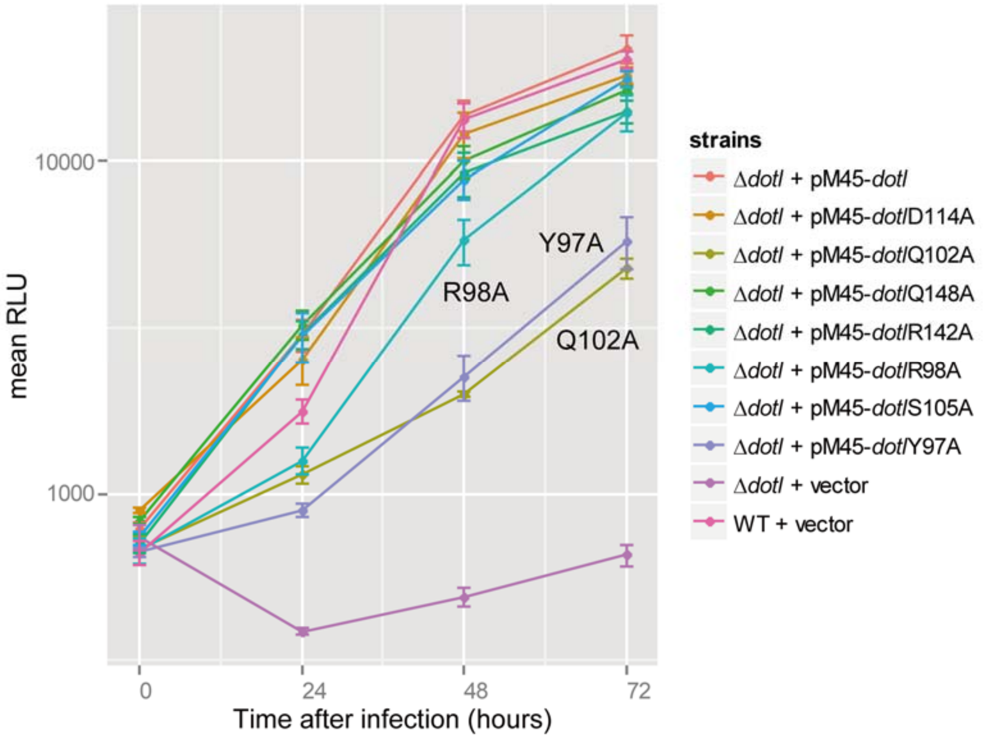

Fig. S3. Intracellular growth assay of *dotI* alanine substitution mutant strains. *Acanthamoeba castellanii* were infected with the indicated luminescent *L. pneumophila* strains (MOI 2). RLUs were measured at the indicated time points.

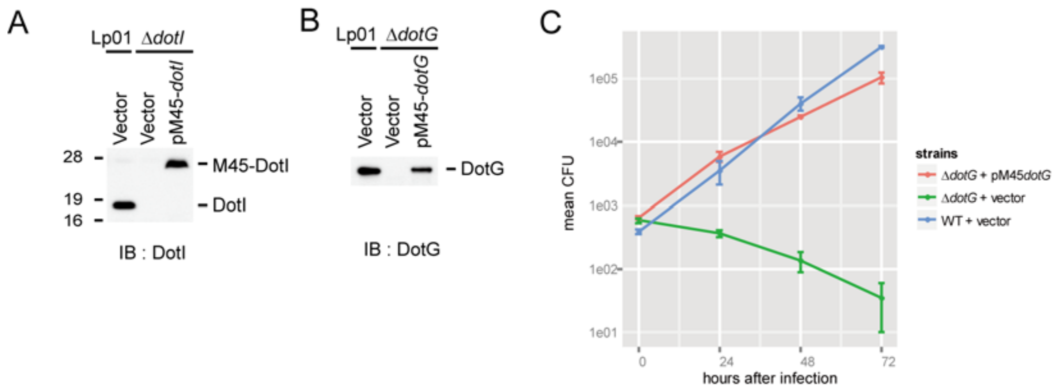

Fig. S4. M45-DotG is functional as authentic DotG. (A) M45-DotI is expressed in *L. pneumophila* at the equivalent level to DotI in wild type. (B) M45-DotG is expressed in *L. pneumophila* at the equivalent level to DotG in wild type. (C) M45-tagging to DotG does not affect intracellular growth in *Acanthamoeba castellanii*.

A

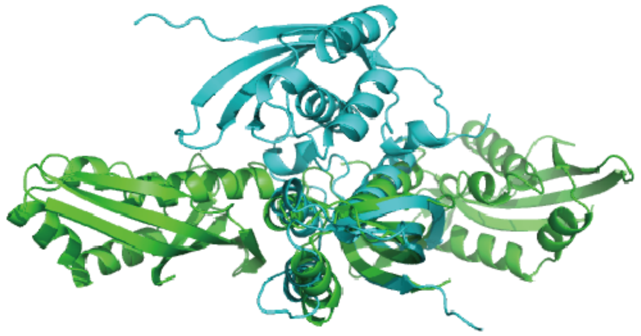

B

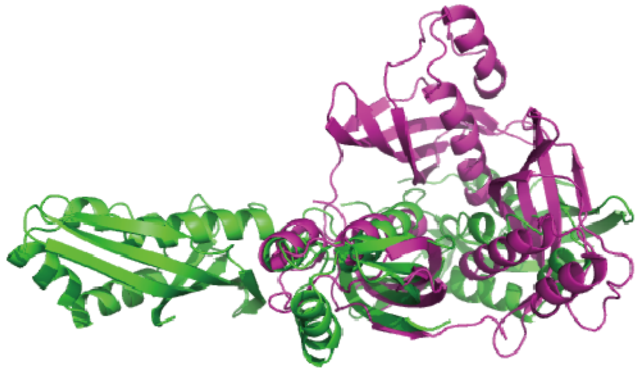

Fig. S5. Protomer arrangements of crystal structures of VirB8 structural homologs are distinct from each other. Arbitrarily chosen protomers in crystal structures of perimplasmic domains of *L. pneumophila* DotI (green), *A. tumefaciens* VirB8 (cyan) and *E. faecalis* TraM (magenta) were superimposed. Neighboring protomers are also shown. (A) *A. tumefaciens* VirB8 (PDB id 2cc3) is superimposed onto DotI. (B) *E. faecalis* TraM (PDB id 4EC6) is superimposed onto DotI.
